# Death of a Drunkard, Once a Physician

**Published:** 1846-02

**Authors:** 


*Death of a Drunkard, once a Physician.*—A short time since, the Rev. Mr. Robinson, of the Episcopal Seaman's Mission, of this city, found a miserable-looking sailor sitting in the street, reading a Greek poet. He was conveyed to Chelsea Hospital, and here is his last history, from the Christian Witness.

"Thursday, very anxious for poor Deven; fear he will not get well; his case has excited much interest wherever I have mentioned it. Born in Philadelphia, of respectable English parents, he was sent, after a preparatory course of study, to Oxford, England, where he graduated in 1819. Thence he went to London, and attended the Medical College, where he received his diploma. His history from that time is mostly involved in mystery. Some six years since, he entered as surgeon in a Portuguese regiment, at Tenior, in the East Indies, which situation he held about five years, when he left to return to the United States. The vessel in which he was a passenger, was wrecked on the Island of Madagascar, when he lost all his effects. From Madagascar he went to the Isle of France, where he shipped as a common sailor, in a bark which arrived at Salem in the middle of November. Found him in a state of extreme destitution; provided him with necessary clothing, and made arrangements for keeping him near me and under my influence a little while, in the hope of his restoration to that position in society, which he was educated to occupy.

"Saturday. To-day heard of the death of Deven. His history, which he promised to write out for me, must now remain a secret. Possessed of superior natural powers, and an education more thorough than most young men of our country, he might have shed lustre on his profession, had not the demon, intemperance, obtained the mastery over him. A native of the same city, and acquainted, as he stated, with me in my childhood, I have felt the most prayerful solicitude to reclaim him if possible; and cannot but think if we had had a 'home,' where he could have been received and carefully watched over, he might have had a longer space for repentance and reformation."—*Baston Med. and Sur. Jour.*

